# Impact of positive chest X-ray findings and blood cultures on adverse outcomes following hospitalized pneumococcal lower respiratory tract infection: a population-based cohort study

**DOI:** 10.1186/1471-2334-13-197

**Published:** 2013-05-02

**Authors:** Marlene Skovgaard, Henrik C Schønheyder, Thomas Benfield, Rikke B Nielsen, Jenny D Knudsen, Jette Bangsborg, Christian Østergaard, Hans-Christian Slotved, Helle Bossen Konradsen, Lotte Lambertsen, Reimar W Thomsen

**Affiliations:** 1 Department of Clinical Epidemiology, Aarhus University Hospital, Aarhus N, 8200, Denmark; 2 Department of Clinical Microbiology, Aalborg University Hospital, Aalborg, 9000, Denmark; 3 Department of Infectious Diseases, Copenhagen University Hospital – Hvidovre, Hvidovre, 2650, Denmark; 4 Department of Clinical Microbiology, Copenhagen University Hospital – Hvidovre, Hvidovre, 2650, Denmark; 5 Department of Clinical Microbiology, Copenhagen University Hospital – Herlev, Herlev, 2730, Denmark; 6 Statens Serum Institut, Copenhagen S, 2300, Denmark

**Keywords:** Streptococcus pneumoniae, Pneumococcal infection/diagnosis, Respiratory tract infection, Pneumonia, Bacteremia, Sepsis, Epidemiologic study, Outcome assessment (Health care), Thoracic radiography

## Abstract

**Background:**

Little is known about the clinical presentation and outcome of pneumococcal lower respiratory tract infection (LRTI) without positive chest X-ray findings and blood cultures. We investigated the prognostic impact of a pulmonary infiltrate and bacteraemia on the clinical course of hospitalized patients with confirmed pneumococcal LRTI.

**Methods:**

We studied a population-based multi-centre cohort of 705 adults hospitalized with LRTI and *Streptococcus pneumoniae* in LRT specimens or blood: 193 without pulmonary infiltrate or bacteraemia, 250 with X-ray confirmed pneumonia, and 262 with bacteraemia. We compared adverse outcomes in the three groups and used multiple regression analyses to adjust for differences in age, sex, comorbidity, and lifestyle factors.

**Results:**

Patients with no infiltrate and no bacteraemia were of similar age but had more comorbidity than the other groups (Charlson index score ≥1: no infiltrate and no bacteraemia 81% vs. infiltrate without bacteraemia 72% vs. bacteraemia 61%), smoked more tobacco, and had more respiratory symptoms. In contrast, patients with a pulmonary infiltrate or bacteraemia had more inflammation (median C-reactive protein: no infiltrate and no bacteraemia 82 mg/L vs. infiltrate without bacteraemia 163 mg/L vs. bacteraemia 316 mg/L) and higher acute disease severity scores. All adverse outcomes increased from patients with no infiltrate and no bacteraemia to those with an infiltrate and to those with bacteraemia: Length of hospital stay (5 vs. 6 vs. 8 days); intensive care admission (7% vs. 20% vs. 23%); pulmonary complications (1% vs. 5% vs. 14%); and 30-day mortality (5% vs. 11% vs. 21%). Compared with patients with no infiltrate and no bacteraemia, the adjusted 30-day mortality rate ratio was 1.9 (95% confidence interval (CI) 0.9-4.1) in patients with an infiltrate without bacteraemia and 4.1 (95% CI 2.0-8.5) in bacteraemia patients. Adjustment for acute disease severity and inflammatory markers weakened these associations.

**Conclusions:**

Hospitalization with confirmed pneumococcal LRTI is associated with substantial morbidity and mortality even without positive chest X-ray findings and blood cultures. Still, there is a clinically important outcome gradient from LRTI patients with pneumococcal isolation only to those with detected pulmonary infiltrate or bacteraemia which is partly mediated by higher acute disease severity and inflammation.

## Background

*Streptococcus pneumoniae* is the most frequently identified pathogen associated with community acquired pneumonia (CAP) [1] and bacterial lower respiratory tract infection (LRTI) [2]. *S. pneumoniae* accounts for an estimated 30% of all hospitalized CAP episodes [3] and for up to half of CAP episodes where aetiology is known [4-8]. The burden of pneumococcal LRTI is likely to increase in Western populations due to the increasing number of elderly citizens with chronic diseases. A recent American study estimated that hospitalizations for pneumococcal pneumonia will increase by 96% from 2004 to 2040, with an amplified annual cost of $2.5 billion in the USA [9]. Thirty-day mortality following hospitalization with pneumococcal pneumonia is 5-13% for non-bacteraemic episodes [10,11], 16-21% for bacteraemic pneumococcal pneumonia [10-13], and 15-37% for episodes in the ICU [14,15].

In-hospital diagnostics usually include chest X-ray and blood culturing, but hospitalized pneumococcal LRTI episodes do not always present themselves with bacteraemia or a pulmonary infiltrate. Presence of bacteraemia or a pulmonary infiltrate without bacteraemia in patients with pneumococcal LRTI may be associated with a worse prognosis due to higher acute disease severity, inflammation and hypoxaemia [16], but data on this association are limited. A few studies of limited size have compared the prognosis of pneumococcal pneumonia and bacteraemia respectively, with conflicting results [10,14,17,18]. Others compared ‘all-cause’ LRTI patients with and without pulmonary infiltrates, and showed a statistically non-significantly increased mortality associated with the presence of an infiltrate [19-21]. However, little is known about the presentation and outcome of hospitalized pneumococcal LRTI with no pulmonary infiltrate and no bacteraemia.

To improve clinical surveillance and treatment of pneumococcal infections, it is important to improve our understanding of the disease pathogenesis and the clinical course of the entire spectrum of pneumococcal LRTI among hospitalized patients. This includes clarifying whether X-ray findings and blood cultures add extra information on the clinical course and consequences of confirmed pneumococcal infection, or if they are merely of diagnostic value or of importance to researchers. We therefore undertook a population-based study covering one-third of Denmark’s population to examine clinical presentation and outcomes among three manifestation groups of pneumococcal LRTI, hypothesizing a clinical outcome gradient from patients with no infiltrate and no bacteraemia to those with an infiltrate without bacteraemia to those with detected bacteraemia.

## Methods

### Study population

From January 1^st^ 2011 to December 31^st^ 2011 a population based cohort was created, including patients over the age of 15 years with a pneumococcal isolate from the lower respiratory tract and/or the blood diagnosed by one of three Danish Departments of Clinical Microbiology at Aalborg Hospital, Herlev Hospital and Hvidovre Hospital, respectively. The total uptake area for the three departments was approximately two million people (~35% of Denmark’s 5.6 million inhabitants), providing microbiological services for patients from 15 hospitals and over 1000 general practitioners in that area.

A detailed chart review was done for 1,169 episodes with a positive culture for *S. pneumoniae*, to access information on LRTI diagnosis and its manifestation (site of pneumococcal isolation and chest X-ray findings), patient and clinical characteristics, and outcomes. Because the focus of our study was prognosis following incident hospitalization with acute pneumococcal LRTI, we excluded episodes treated in the primary care setting only (n=327), repeated hospitalizations with pneumococcal LRTI during 2011 (n=34), and patients with no LRT focus, i.e.; neither an acute LRTI discharge diagnosis (ICD10 codes: J13, J18.0, J18.1, J18.2, J18.8, J18.9, J20, J20.2, J20.9, J22, J44.0, J44.1) nor any new or worsened LRTI symptoms (cough, sputum production, dyspnoea, chest pain), nor any new pulmonary infiltrate (n=63). Among patients with no pulmonary infiltrate and no pneumococcal bacteraemia, we further excluded patients without elevated inflammatory markers (C-reactive protein (CRP) >50 mg/L and/or leukocyte count >8.8*10^9^/L) and at least one LRTI symptom (n= 35), to maximise the likelihood of acute pneumococcal infection and not merely colonization. Patients without a valid Danish Civil Registration Number (i.e. individuals not living permanently in Denmark) were also excluded (n= 5). Thus, the final study cohort consisted of 705 hospitalized patients aged 15 years or older with acute LRTI and a pneumococcal isolate from the LRT (n=443) and/or blood (n=262) (see flow chart in Additional file 1).

### Exposure: pneumococcal LRTI manifestation

We divided our patient cohort into three predefined pneumococcal LRTI manifestation groups, based on pneumococcal isolation site and chest X-ray findings: pneumococcal bacteraemia, pulmonary infiltrate without bacteraemia, and neither pulmonary infiltrate nor bacteraemia. Patients with pneumococcal isolates from both blood and LRT (n=55) were included in the bacteraemia group. Patients from whom no blood culture had been obtained within four days from the LRT sample (n=152) or who had no chest X-ray taken within seven days from the index date (n=71) were considered to have no bacteraemia or no infiltrate, respectively. The index date was defined as the LRT or blood specimen sample date. For the 55 patients (8%) who had both a positive blood culture and a positive LRT sample, we defined the index date as that closest to the date of chest X-ray or, in the absence of an X-ray (n=6), as the date of the first positive sample. X-ray findings were recorded from the written interpretation provided by the radiologist and/or from the medical records.

We registered results for all sputum Gram stains, cultures of LRT samples, blood cultures, and pleural fluid cultures as obtained at the managing physician’s discretion (*i.e.,* when infection was suspected). The vast majority of the LRT samples were sputum (80%), 15% of samples were obtained by blind endotracheal suction, whereas the remaining 5% were BAL or pleural fluid (see Additional file 2). The methods for performing each of these tests were those in place at the participating institutions, however, at all departments sputum was processed for culture only if it contained a significant number of leukocytes (>25 per low power field) with or without columnar epithelial cells, and/or no or few squamous epithelial cells (<10 per low power field) [22,23]. Blood samples were cultured for at least five days before declared negative. A sample was only regarded as positive for pneumococci if a culture isolate was available. Identification of *S. pneumoniae* was done with standard methods including Gram stain, detection of capsular antigen by latex agglutination or quelling test as well as optochin test or bile solubility test [24,25].

### Preadmission patient and clinical characteristics

We ascertained important preadmission patient characteristics that may be associated with both the type of pneumococcal LRTI manifestation and the patient’s risk of death, including age, sex, comorbidity, smoking, and alcohol overuse [12,13,26-29]. We also recorded body mass index (BMI), travel history, housing, and pneumococcal vaccination status if noted in the medical records.

To account for comorbidity as a potential confounder, Charlson’s comorbidity index scores were calculated for each patient. The Charlson index is a weighted index that includes 19 different disease categories and takes into account the number and the seriousness of comorbid disease [12,13,30]. Data on each patient’s complete hospitalization history of co-existing diseases was obtained from The Danish National Registry of Patients (DNRP) using linkage by unique civil registration numbers. The DNRP includes all patient admissions to Danish hospitals since 1977 and all hospital outpatient visits since 1995 [31]. Three comorbidity levels were defined: low (score of 0), medium (1–2), and high (≥3).

We also examined clinical presentation and acute symptoms within 24 hours of the index date. We ascertained presence of acute respiratory symptoms (dyspnoea, cough, sputum production or chest pain), plasma glucose level, CRP level, leukocyte count, body temperature, mean arterial blood pressure (MAP), peripheral oxygen saturation (SAT), and two disease severity scores; the CURB-65 score [32] and the Pitt score [33].

### Outcomes

The primary endpoint was all-cause 30-day mortality following the index date. Complete follow-up for mortality was ensured by linkage to the Danish Civil Registration System (CRS), which encompasses all residents who live – or have lived – in Denmark since 1968 [34].

Secondary endpoints were length of hospital stay (LOS), admission to an intensive care unit (ICU), and pulmonary complications (ICD-10 codes of pleural effusion J90 and J91, pleural drainage KGAA96, KGAA97, KGAA10, empyema J86, lung abscess J85, or ARDS J80.9). If a patient was discharged and readmitted again within 48 hours, total LOS was calculated from the date of admission to the day of the latest discharge.

### Statistical analyses

For the three LRTI manifestation groups, we compared the distribution of baseline and clinical characteristics and calculated prevalence proportion ratios with 95% confidence intervals (CIs) and medians with interquartile range (IQR) as displayed in Tables 1 and 2. We examined the association between LRTI manifestation group and the risk of intensive care admission and pulmonary complications by Poisson regression. We used linear regression to examine the ratios of the means of LOS in the three groups. For mortality analyses we constructed Kaplan-Meier survival curves and computed cumulative mortality. Cox’s regression was used to compute 30-day mortality rate ratio (MRR) estimates with 95% CIs comparing mortality for the three manifestation groups. The regression analyses were used to control for predefined major potential confounders: sex, age (as a continuous variable), level of comorbidity, current or former smoking, and excessive alcohol consumption (>7 units per week for women and >14 units per week for men). We also performed an additional adjusted analysis to assess whether differences in acute disease severity and inflammatory markers accounted for differences in 30-day mortality.

We analysed data using Stata software (version 11.2; StataCorp, College Station, TX, USA). The National Health Board (record no. 7-604-04-2/332) and the Danish Data Protection Agency (record no. 2011-41-6426) approved this study.

**Table 1:** Preadmission baseline characteristics of 705 patients hospitalized for pneumococcal LRTI, according to manifestation group

**Characteristics**		**No infiltrate and no bacteraemia patients**		**Infiltrate without bacteraemia patients**		**Bacteraemia patients**	
		**n (%) **^**a**^	**PR (95% CI)**	**n (%) **^**a**^	**PR (95% CI)**	**n (%) **^**a**^	**PR (95% CI)**
All patients		193 (100)		250 (100)		262 (100)	
Age (years)							
	Median	68 (59–76)		69 (57–79)		68 (57–80)	
	15-49	22 (11)	1.0 (ref.)	38 (15)	1.3 (0.8-2.2)	47 (18)	1.6 (1.0-2.5)
	50-74	116 (60)	1.0 (ref.)	129 (52)	0.9 (0.7-1.0)	128 (49)	0.8 (0.7-1.0)
	≥75	55 (29)	1.0 (ref.)	83 (33)	1.2 (0.9-1.5)	87 (33)	1.2 (0.9-1.5)
Sex							
	Women	83 (43)	1.0 (ref.)	90 (36)	0.8 (0.7-1.1)	123 (47)	1.1 (0.9-1.3)
	Men	110 (57)	1.0 (ref.)	160 (64)	1.1 (1.0-1.3)	139 (53)	0.9 (0.8-1.1)
Charlson comorbidity Index							
	Index low (0)	36 (19)	1.0 (ref.)	70 (28)	1.5 (1.1-2.1)	101 (39)	2.1 (1.5-2.9)
	Index medium (1–2)	96 (50)	1.0 (ref.)	92 (37)	0.7 (0.6-0.9)	81 (31)	0.6 (0.5-0.8)
	Index high (≥3)	61 (32)	1.0 (ref.)	88 (35)	1.1 (0.9-1.5)	80 (31)	1.0 (0.7-1.3)
Chronic pulmonary disease ^b^		112 (58)	1.0 (ref.)	114 (46)	0.8 (0.7-0.9)	58 (22)	0.4 (0.3-0.5)
Chronic heart disease ^c^		26 (13)	1.0 (ref.)	35 (14)	1.0 (0.6-1.7)	35 (13)	1.0 (0.6-1.6)
BMI (median)		23.5 (20.0-27.4)		23.3 (21.0-26.3)		23.6 (21.9-26.7)	
Smoking		112 (58)	1.0 (ref.)	114 (46)	0.8 (0.7-0.9)	58 (22)	0.4 (0.3-0.5)
	Yes, currently	106 (58)	1.0 (ref.)	122 (53)	0.9 (0.8-1.1)	99 (43)	0.7 (0.6-0.9)
	No, former	57 (31)	1.0 (ref.)	69 (30)	1.2 (0.6-2.4)	66 (29)	3.1 (1.7-5.7)
	No, never	12 (7)	1.0 (ref.)	18 (8)	1.0 (0.7-1.3)	47 (20)	0.9 (0.7-1.2)
	No, unknown	7 (4)	1.0 (ref.)	21 (9)	2.4 (1.0-5.5)	18 (8)	2.0 (0.8-4.8)
Alcohol							
	>7/14 per week ^d^	31 (18)	1.0 (ref.)	60 (27)	1.5 (1.0-1.7)	48 (23)	1.2 (0.8-1.9)
	≤7/14 per week ^d^	68 (40)	1.0 (ref.)	89 (40)	1.0 (0.8-1.3)	69 (32)	0.8 (0.6-1.1)
	Never	71 (42)	1.0 (ref.)	76 (34)	0.8 (0.6-1.0)	96 (45)	1.1 (0.9-1.4)
Employment							
	Employed	18 (12)	1.0 (ref.)	24 (13)	1.0 (0.6-1.8)	47 (24)	1.9 (1.2-3.2)
	Unemployed	7 (5)	1.0 (ref.)	10 (5)	1.1 (0.4-2.9)	5 (3)	0.5 (0.2-1.6)
	Pension	117 (81)	1.0 (ref.)	142 (76)	0.9 (0.8-1.1)	140 (70)	0.9 (0.8-1.0)
	Other ^e^	3 (2)	1.0 (ref.)	10 (5)	2.6 (0.7-9.3)	6 (3)	1.5 (0.4-5.8)
Housing							
	Own house/apartment	166 (94)	1.0 (ref.)	207 (89)	1.0 (0.9-1.0)	213 (93)	1.0 (0.9-1.0)
	Institution	9 (5)	1.0 (ref.)	20 (9)	1.7 (0.8-3.6)	14 (6)	1.2 (0.5-2.7)
	Homeless	2 (1)	1.0 (ref.)	5 (2)	1.9 (0.4-9.7)	1 (0)	0.4 (0.0-4.2)
Travel history ^f^		7 (4)	1.0 (ref.)	7 (3)	0.8 (0.3-2.2)	7 (3)	0.7 (0.3-2.1)
Vaccination							
	Influenza vaccine	2 (1)	1.0 (ref.)	1 (0)	NA	0 (0)	NA
	Pneumococcal vaccine	0 (0)	1.0 (ref.)	1 (0)	NA	0 (0)	NA

LRTI, lower respiratory tract infection; PR, prevalence ratio; CI, confidence interval; ref, reference; NA, not applicable; BMI, Body mass index.

^a^ Data are n (% of everyone we have data for) or median (interquartile range).

^b^ Chronic pulmonary disease: ICD-8: 490–493, 515–518. ICD-10: DJ40-DJ47, DJ60-DJ67, DJ68.4, DJ70.1, DJ70.3, DJ84.1, DJ92.0, DJ96.1, DJ98.2, DJ98.3.

^c^ Congestive heart failure: ICD-8: 427.09, 427.10, 427.11, 427.19, 428.99, 782.49. ICD-10: DI50, DI11.0, DI13.0, DI13.2.

^d^ 7 units of alcohol for women and 14 for men per week.

^e^ “Other” employment covers: students, maternity leave, and rehabilitation support.

^f^ Travel within three month prior to the index date.

Results were available for >87% except for BMI (could be calculated for 20-26% of patients), employment (available for 74-75%), alcohol use (81-90%) and vaccination (only noted if defiantly positive).

**Table 2:** Clinical characteristics +/−24 hours from the index date according to LRTI manifestation group

**Characteristics**		**No infiltrate and no bacteraemia patients**		**Infiltrate without bacteraemia patients**		**Bacteraemia patients**	
		**n (%) **^**a**^	**PR (95% CI)**	**n (%) **^**a**^	**PR (95% CI)**	**n (%) **^**a**^	**PR (95% CI)**
Medical ward		188 (97)	1.0 (ref.)	229 (92)	0.9 (0.9-1.0)	249 (95)	1.0 (0.9-1.0)
Community acquired ^b^		167 (87)	1.0 (ref.)	217 (87)	1.0 (0.9-1.1)	242 (92)	1.1 (1.0-1.1)
Antibiotics		155 (80)	1.0 (ref.)	235 (94)	1.2 (1.1-1.3)	260 (99)	1.2 (1.2-1.3)
Aetiology							
	Pneumococcal only	137 (71)	1.0 (ref.)	178 (71)	1.0 (0.9-1.1)	219 (84)	1.2 (1.1-1.3)
	Pneumococcal mixed ^c^	56 (29)	1.0 (ref.)	72 (29)	1.0 (0.7-1.3)	43 (16)	0.6 (0.4-0.8)
Respiratory symptoms ^d^							
	0	0 (0)	1.0 (ref.)	20 (8)	NA	35 (13)	NA
	1	33 (17)	1.0 (ref.)	25 (10)	0.6 (0.4-0.9)	56 (21)	1.3 (0.8-1.8)
	2	44 (23)	1.0 (ref.)	59 (24)	1.0 (0.7-1.5)	79 (30)	1.3 (1.0-1.8)
	≥3	116 (60)	1.0 (ref.)	146 (58)	1.0 (0.8-1.1)	92 (36)	0.6 (0.5-0.7)
Oxygen saturation (peripheral)		94 (91–96)		92 (88–95)		93 (89–95)	
Temperature ≥38.5°C		35 (20)	1.0 (ref.)	86 (37)	1.8 (1.3-2.6)	126 (50)	2.5 (1.8-3.4)
MAP (mmHg)		91 (78–103)		88 (73–99)		80 (72–97)	
CURB65 ≥2		68 (35)	1.0 (ref.)	121 (48)	1.4 (1.1-1.7)	160 (61)	1.7 (1.4-2.1)
PITT ≥4		12 (6)	1.0 (ref.)	28 (11)	1.8 (0.9-3.4)	41 (16)	2.5 (1.4-4.7)
CRP (mg/L)							
	Median	82 (36–140)		163 (70–246)		316 (219–404)	
	<50	54 (31)	1.0 (ref.)	41 (17)	0.6 (0.4-0.8)	8 (3)	0.1 (0.1-0.2)
	50-99	46 (26)	1.0 (ref.)	36 (15)	0.6 (0.4-0.9)	14 (6)	0.2 (0.1-0.4)
	100-199	52 (30)	1.0 (ref.)	68 (29)	1.0 (0.7-1.3)	32 (13)	0.4 (0.3-0.6)
	≥200	24 (14)	1.0 (ref.)	91 (39)	2.8 (1.9-4.2)	196 (78)	5.7 (3.9-8.4)
Leukocyte count (10^9^/L)							
	Median	14 (11–17)		14 (11–19)		17 (12–23)	
	<3,5	3 (1)	1.0 (ref.)	8 (3)	6.1 (0.8-48.0)	17 (7)	12.2 (1.6-90.5)
	3.5-8.7	17 (10)	1.0 (ref.)	25 (11)	1.1 (0.6-2.0)	22 (9)	0.9 (0.5-1.7)
	8.8-14.9	90 (51)	1.0 (ref.)	98 (42)	0.8 (0.7-1.0)	57 (23)	0.5 (0.3-0.6)
	≥15.0	70 (40)	1.0 (ref.)	103 (44)	1.1 (0.9-1.4)	153 (61)	1.6 (1.3-1.9)

LRTI, lower respiratory tract infection; PR, prevalence ratio; CI, confidence interval; ref, reference; NA, not applicable; MAP, Mean Arterial Pressure; CURB65, Confusion Urea Respiration-frequency Blood-pressure 65years; PITT , Pitt’s bacteraemia severity score; CRP, C-reactive protein.

^a^ In the n (%) columns data are either n (% of everyone we have data for) or median (interquartile range).

^b^ Community acquired infections are defined as having the index date ≤ 2 days form admission.

^c^ Mixed aetiology is defined as finding of other pathogenic bacteria than pneumococci in either blood or LRT specimen.

^d^ Respiratory symptoms include dyspnoea, cough, sputum production and chest pain.

Results were available for >87% of the patients except the individual measures that constitute the CURB65 score (58-99% worst for respiratory frequency and carbamide). CURB65 and PITT were calculated out of everyone, though at least one measure pr. patient was missing in 60-62% of patients for the CURB65 score and 9-18% for the PITT score. Missing valuables were regarded as “no”. Sums were thus calculated as “minimums”.

## Results

### Descriptive data

Of the 705 adult in-patients with confirmed acute pneumococcal LRTI, 262 (37%) had pneumococcal bacteraemia. Of the 443 without bacteraemia, 250 (56%) had X-ray confirmed pneumonia and 193 (44%) had neither bacteraemia nor a pulmonary infiltrate. The age was similar between groups: 68 years (IQR 59–76) among patients with no infiltrate and no bacteraemia, 69 years (57–79) among patients with an infiltrate and no bacteraemia and 68 years (57–80) for bacteraemia patients. Sex, BMI, alcohol intake, housing, travel history, and vaccination status did not differ materially by clinical manifestation of pneumococcal disease (Table 1). Patients with pneumococcal LRTI with no infiltrate and no bacteraemia had more chronic pulmonary disease and more overall comorbidity than the other groups (Charlson index score ≥1: no infiltrate and no bacteraemia 81% vs. infiltrate without bacteraemia 72% vs. bacteraemia 61%), and they smoked more tobacco. Based on our chart review and discharge diagnoses, patients with no infiltrate and no bacteraemia often had acute bronchitis without pneumonia, exacerbation of chronic obstructive lung disease, or suspected clinical pneumonia where the timing of the chest X-ray was suboptimal compared with the time of peak clinical LRTI symptoms (see Additional file 3).

Table 2 provides data on clinical characteristics within 24 hours from the index date. The vast majority of patients were acutely admitted to a medical ward (92-97%), had community acquired infection (87-92%), and had no additional bacterial pathogens in their blood or lower airways (71-84%). Patients with no infiltrate and no bacteraemia were less likely to receive antibiotic treatment on the index date of obtaining the sample or have a fever, while they had more respiratory symptoms than the bacteraemia patients. The most prevalent symptom for all groups was cough (65-80%, lowest for the bacteraemia group). Eight per cent of patients in the no infiltrate and no bacteraemia group, versus 1% in both the infiltrate without bacteraemia and the bacteraemia groups did not receive antibiotics at any point of time during hospitalization. CRP rose from 82 mg/L in the no infiltrate and no bacteraemia group to 163 in the infiltrate without bacteraemia group and 316 in the bacteraemia group, whereas counts of leukocytes rose from 14*10^9^/L (IQR: 11–17) to 14 (11–19) to 17 (12–23), respectively (Table 2). Stratification by age revealed similar successive increases in CRP and leukocyte counts across LRTI manifestations in all age groups (see Additional file 4).

Data on plasma glucose levels were available for 60-71% of the patients and showed that the fraction of patients with glucose above 11.1 mmol/L rose from 10% in the no infiltrate and no bacteraemia group to 15% and 18% in the infiltrate without bacteraemia and bacteraemia groups, respectively. Of the patients with no infiltrate and no bacteraemia, 55% received a discharge diagnosis of acute LRTI, compared with 72% and 76% of patients with infiltrate but no bacteraemia and bacteraemia, respectively (see Additional file 3).

### Pneumococcal LRTI manifestation and 30-day mortality

**Figure 1:**
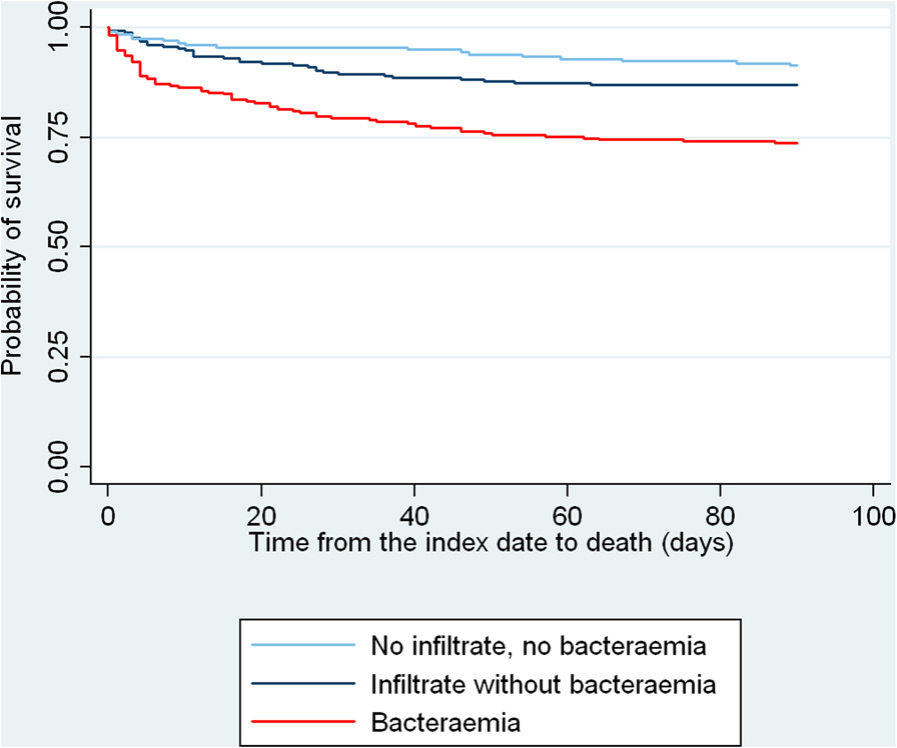
**Kaplan-Meier survival curves for patients hospitalized with pneumococcal LRTI according to manifestation group.** The numbers at risk represent the number of patients still at risk of death at a given time during the follow-up period. LRTI, lower respiratory tract infection.

Thirty-day mortality among patients with no infiltrate and no bacteraemia reached 5%, as compared with 11% and 21% for patients with an infiltrate without bacteraemia and patients with bacteraemia, respectively. When dividing the bacteraemia group further into those with (202/262=77%) and without (60/262=23%) a pulmonary infiltrate, mortality was highest for bacteraemia without an infiltrate (28 vs. 18%). As seen from Figure 1, patients with bacteraemia had higher mortality than the other patients from the first days of observation; whereas the two patient groups without bacteraemia had rather similar mortality during the first week after which a difference between the two became more apparent. Compared with patients with no infiltrate and no bacteraemia, the adjusted 30-day MRR was 1.9 (95% CI 0.9-4.1) in patients with an infiltrate without bacteraemia and 4.1 (2.0-8.5) in bacteraemia patients (Table 3). Supplementary analyses showed adjusted 7-day MRRs of 1.1 (0.4-3.1) for the infiltrate without bacteraemia group and 4.0 (1.7-9.9) for the bacteraemia group, respectively, and 8-30-day MRRs of 3.5 (1.0-12.5) and 4.2 (1.2-14.8). A similar 30-day mortality gradient from patients with no infiltrate and no bacteraemia, over those with infiltrate without bacteraemia, to bacteraemia patients was observed for males and females, and in different patient groups according to age and comorbidity level (Table 3). Stratification for level of CRP revealed that the association between LRTI manifestation group and mortality weakened with rising CRP, *i.e*., the prognostic impact of pulmonary infiltrate and bacteraemia was less pronounced among patients who all had high CRP levels (Table 3). The additional adjusted analysis showed that further inclusion of the acute disease markers CRP, leukocyte count, MAP and SAT in the regression model reduced the 30-day MRR for infiltrate without bacteraemia and bacteraemia patients to 1.6 (0.7-3.6) and 2.9 (1.2-7.0) respectively.

**Table 3:** 30-day-mortality according to LRTI manifestation group overall and stratified by subgroups

**Prognostic factor**		**No infiltrate and no bacteraemia patients**			**Infiltrate without bacteraemia patients**			**Bacteraemia patients**		
		**n (%)**	**Crude MRR (95 % CI) **^**a**^	**Adjusted MRR (95% CI) **^**a,b**^	**n (%)**	**Crude MRR (95% CI) **^**a**^	**Adjusted MRR (95% CI) **^**a,b**^	**n (%)**	**Crude MRR (95 % CI) **^**a**^	**Adjusted MRR (95% CI) **^**a,b**^
All patients		9 (5)	1.0 (ref.)	1.0 (ref.)	27 (11)	2.4 (1.1-5.0)	1.9 (0.9-4.1)	54 (21)	4.8 (2.4-9.6)	4.1 (2.0-8.5)
Age group (years)										
	15-49	0 (0)	1.0 (ref.)	1.0 (ref.)	3 (8)	NA	NA	2 (4)	NA	NA
	50-74	5 (4)	1.0 (ref.)	1.0 (ref.)	13 (10)	2.3 (0.8-6.6)	2.1 (0.7-6.0)	28 (22)	5.5 (2.1-14.3)	5.2 (2.0-13.8)
	≥75	4 (7)	1.0 (ref.)	1.0 (ref.)	11 (13)	1.8 (0.6-5.8)	1.6 (0.5-5.3)	24 (28)	4.1 (1.4-11.9)	3.3 (1.1-9.9)
Sex										
	Women	3 (4)	1.0 (ref.)	1.0 (ref.)	7 (8)	2.2 (0.6-8.3)	2.2 (0.6-9.0)	20 (16)	4.7 (1.4-15.9)	4.3 (1.2-15.6)
	Men	6 (5)	1.0 (ref.)	1.0 (ref.)	20 (13)	2.3 (0.9-5.8)	1.8 (0.7-4.5)	34 (24)	4.9 (2.1-11.7)	4.2 (1.7-10.3)
Comorbidity index										
	Index low (0)	1 (3)	1.0 (ref.)	1.0 (ref.)	4 (6)	2.1 (0.2-18.6)	1.5 (0.2-14.7)	15 (15)	5.7 (0.7-42.8)	4.1 (0.5-32.5)
	Index medium (1–2)	2 (2)	1.0 (ref.)	1.0 (ref.)	13 (14)	7.1 (1.6-31.5)	5.8 (1.3-26.2)	14 (17)	8.9 (2.0-39.1)	8.1 (1.8-36.8)
	Index high (≥3)	5 (9)	1.0 (ref.)	1.0 (ref.)	10 (12)	1.1 (0.4-3.1)	0.8 (0.3-2.3)	25 (31)	3.5 (1.4-8.5)	2.9 (1.2-7.3)
Chronic pulmonary disease ^c^										
	Yes	6 (5)	1.0 (ref.)	1.0 (ref.)	12 (11)	2.0 (0.7-5.3)	1.3 (0.5-3.5)	14 (24)	4.8 (1.9-12.6)	3.8 (1.4-10.3)
	No	3 (4)	1.0 (ref.)	1.0 (ref.)	15 (11)	3.1 (0.9-10.5)	2.6 (0.7-9.4)	40 (20)	5.7 (1.8-18.6)	4.5 (1.4-14.9)
CRP (mg/L)										
	<100	1 (1)	1.0 (ref.)	1.0 (ref.)	8 (10)	10.7 (1.3-85.8)	11.3 (1.4-95.4)	7 (32)	35.6 (4.4-290)	27.6 (2.8-267)
	100-199	3 (6)	1.0 (ref.)	1.0 (ref.)	6 (9)	1.5 (0.4-6.1)	1.1 (0.2-5.3)	4 (13)	2.2 (0.5-9.6)	2.8 (0.5-15.7)
	≥200	4 (17)	1.0 (ref.)	1.0 (ref.)	12 (13)	0.8 (0.3-2.4)	0.6 (0.2-2.1)	41 (21)	1.3 (0.5-3.6)	1.3 (0.5-3.6)
Leukocyte count (10^9^/L)										
	<3,5	1 (100)	1.0 (ref.)	1.0 (ref.)	3 (38)	0.1 (0.0-2.1)	NA	8 (47)	0.3 (0.0-2.3)	0.2 (0.0-3.4)
	3.5-8.7	0 (0)	1.0 (ref.)	1.0 (ref.)	1 (4)	NA	NA	7 (32)	NA	NA
	8.8-14.9	3 (3)	1.0 (ref.)	1.0 (ref.)	12 (12)	3.7 (1.1-13.3)	2.8 (0.7-10.4)	8 (14)	4.4 (1.2-16.4)	2.7 (0.7-11.6)
	≥15.0	4 (6)	1.0 (ref.)	1.0 (ref.)	10 (10)	1.7 (0.5-5.4)	1.3 (0.4-4.4)	29 (19)	3.5 (1.2-10.0)	3.2 (1.1-9.5)

LRTI, lower respiratory tract infection; MRR, mortality rate ratio; CI, confidence interval; ref, reference; NA, not applicable because of too few events to calculate MRRs; CRP, C-reactive protein.

^a^ MRR estimates are calculated using cox-regression.

^b^ MRR estimates are adjusted for age, sex, comorbidity, smoking, and alcohol intake (except when stratified by variable).

^c^ Chronic pulmonary disease: ICD-8: 490–493, 515–518. ICD-10: DJ40-DJ47, DJ60-DJ67, DJ68.4, DJ70.1, DJ70.3, DJ84.1, DJ92.0, DJ96.1, DJ98.2, DJ98.3.

### Pneumococcal LRTI manifestation and length of stay, ICU admission and pulmonary complications

Table 4 provides data on secondary outcomes. Median length of stay was 5 (IQR 2–9), 6 (4–16) and 8 (4–19) days for patients with no infiltrate and no bacteraemia, infiltrate without bacteraemia, and bacteraemia in that order. Stratification by age groups and level of comorbidity did not reveal any major differences; and similar differences for length of stay were found with exclusion of in-hospital deaths (data not shown). Seven per cent of no infiltrate and no bacteraemia patients were admitted to the ICU during their hospitalization, compared to 20% and 23% among the other two groups. Pulmonary complications occurred in 1% of patients with no infiltrate and no bacteraemia, compared with 5% and 14% among infiltrate without bacteraemia and bacteraemia patients respectively (Table 4). After adjustment for sex, age, comorbidity, smoking, and alcohol consumption, adjusted RRs remained substantially increased for all adverse outcomes among patients with an infiltrate without bacteraemia and patients with bacteraemia, as compared with the no infiltrate and no bacteraemia reference group.

**Table 4:** Length of stay, risk of ICU admission and pulmonary complications according to LRTI manifestation group

	**No infiltrate and no bacteraemia patients**			**Infiltrate without bacteraemia patients**			**Bacteraemia patients**		
	**n (%) **^**a**^	**Crude RR **^**b**^	**Adjusted RR **^**c**^	**n (%) **^**a**^	**Crude RR **^**b**^	**Adjusted RR **^**c**^	**n (%) **^**a**^	**Crude RR **^**b**^	**Adjusted RR **^**c**^
LOS (days)	5 (2–9)	1.0 (ref.)	1.0 (ref.)	6 (4–16)	1.6 (1.3-2.0)	1.5 (1.2-1.9)	8 (4–19)	2.0 (1.6-2.4)	2.1 (1.7-2.6)
ICU admission	14 (7)	1.0 (ref.)	1.0 (ref.)	51 (20)	2.8 (1.6-5.0)	2.4 (1.4-4.1)	60 (23)	3.2 (1.8-5.5)	3.1 (1.8-5.5)
Pulmonary complications ^d^	2 (1)	1.0 (ref.)	1.0 (ref.)	12 (5)	4.6 (1.0-20.5)	4.6 (1.0-21.4)	36 (14)	13.3 (3.2-54.5)	12.6 (2.9-53.8)

LRTI, lower respiratory tract infection; RR, relative risk; LOS, length of stay; ICU, intensive care unit; ref, reference.

^a^ Data are median (interquartile range) or n (% of all in the manifestation group).

^b^ The crude measures are median ratios (95% CI) or prevalence proportion ratios (95% CI).

^c^ The multiple adjusted relative risks are computed using linear regression for LOS, and Poisson regression for ICU admission and pulmonary complications. The estimates are adjusted for age, sex, smoking, alcohol, and comorbidity.

^d^ Pulmonary complications include pleural effusion, pleural drainage, empyema, and lung abscess.

Data available for over 99%.

### Supplementary analyses

To examine potential bias due to misclassification of infection, four sensitivity analyses were made: (1) Because the patient group without infiltrate and no bacteraemia may have included people with chronic pulmonary disease who had pneumococci cultured in the absence of any acute infection due to closer surveillance, we repeated our analyses with restriction to patients who all had CRP >100mg/L or leukocytes >10*10^9^/L together with at least one new or increased pulmonary symptom. This analysis yielded RRs very similar to those in the main analysis (see Additional file 5). (2) We also excluded all patients with chronic pulmonary disease in another analysis, resulting in LOS of 4 vs. 8 vs. 8 days; ICU admission proportions of 8% vs. 29% vs. 23%; pulmonary complications in 2% vs. 7% vs. 14%, and 30-day mortalities of 3% vs. 12% vs. 20%. (3) Third, we reanalysed data while excluding all patients who did not have chest X-rays or blood cultures taken, which also gave similar results as in the main analysis. (4) Finally, we conducted an analysis restricted to patients who had an ICD-10 discharge diagnosis of acute LRTI. This produced robust estimates (Additional file 5).

## Discussion

In this population-based cohort study, presence of a pulmonary infiltrate was associated with a doubled 30-day mortality from pneumococcal LRTI, whereas bacteraemia was associated with a four-fold increased mortality compared with patients with none of these clinical manifestations. Differences in age, comorbidity and lifestyle factors seemed to explain only a minor part of the associations. LOS, risk of ICU admission, and risk of pulmonary complications also rose gradually from patients with no infiltrate and no bacteraemia to those with an infiltrate without bacteraemia and to those with bacteraemia. Non-bacteraemia patients had more acute LRTI symptoms compared to bacteraemia patients, whereas other markers of disease severity (including CRP, leukocyte count, fever, and CURB65 score) gradually increased from patients with no infiltrate and no bacteraemia to those with an infiltrate without bacteraemia to those with detected bacteraemia.

Biological mechanisms underlying the poorer outcome among patients with X-ray changes and bacteraemia may include hypoxaemia due to thickening of the blood-gas barrier and loss of functional residual capacity in the lungs, and increased risk of severe sepsis with decreased perfusion of vital organs. Inflammatory exudate filling alveoli during pneumonia causes a volume loss that impact on lung capacity roughly proportional to the extent of the pulmonary infiltrate [35]. A study of 48 pneumonia patients from Japan found a significant correlation between the size of X-ray pneumonic infiltrates and Pa0_2_ both in young and elderly patients [16]. Hagaman et al. [20] suggested the magnitude of the inflammatory response in the lung tissue to be the most important determinant whether a patient presents with a pulmonary infiltrate or not. Both Hagaman et al. and Basi et al. [19,20] found lower leukocyte counts for patients with negative chest X-rays, corroborating our results. Our findings that additional inclusion of CRP, leukocyte count, MAP and SAT in the regression model reduced the MRRs for both infiltrate without bacteraemia and bacteraemia patients suggest that some of the increased mortality associated with these manifestations is mediated through increased inflammation, hypoxaemia and sepsis.

In the American PORT study based on 2,287 in- and out- patients with CAP of whom 158 had confirmed pneumococcal pneumonia, Brandenburg et al. [17] also noted a tendency towards greater clinical severity for bacteraemic compared with non-bacteraemic patients, as reflected by increased median LOS (7.5 days vs. 6.5 days), pneumonia-related 30-day-mortality (7.7% vs. 2.7%), and ICU admission (15.4% vs. 12.2%) (p-values between 0.4-0.8). In line with this, Musher et al. [10] found bacteraemia to be indicative of a higher 30-day mortality (21 vs. 13%; OR 1.88, p-value 0.25) and higher ICU admission risk (44 vs. 25%; OR 2.38, p-value 0.05) among 100 American veteran patients with pneumococcal pneumonia. Likewise, a Spanish study comprising 57 bacteraemic and 25 non-bacteraemic hospitalized pneumococcal CAP patients found a higher mortality and LOS among bacteraemic patients [18]. In contrast, a South African ICU-study of 63 severe pneumococcal CAP patients found lower in-hospital mortality in bacteraemic compared to non-bacteraemic patients (15% vs. 28%) [14]. In comparison, a sub-analysis of our ICU patients showed a 30-day mortality of 29, 33 and 42% for patients with no infiltrate and no bacteraemia, infiltrate without bacteraemia, and bacteraemia, respectively.

To our knowledge no previous studies have focused on outcomes of pneumococcal LRTI without bacteraemia or pulmonary infiltrate, but studies on unspecified “clinical pneumonia” or “infiltrate-negative all-cause CAP” have been conducted. In a population-based Canadian study of 2706 hospitalised patients Basi et al. [19] reported that approximately one-third of patients suspected of CAP on admission did not have an infiltrate on their admission chest X-ray. The in-hospital mortality was 10% in patients with X-ray confirmed pneumonia compared with 8% in patients without an infiltrate (p=0.09). In a smaller American study of CAP, Hagaman et al. [20] found that patients with presence of an infiltrate stayed one day longer and had slightly higher Pneumonia Severity Index scores and mortality than those with no infiltrate. Similar to our study, Hagaman et al. and Basi et al. did not find any clear association between the amount of pulmonary symptoms and chest X-ray findings.

Our study’s strengths include its relatively large size covering one-third of the Danish population for one year, its population-based design including all hospitals in a geographical region, complete follow-up for mortality, and detailed information on blood chemistry, lifestyle factors, and microbiology data for all patients, thus allowing for extensive adjustment for important confounders and investigation of mediators. Use of routinely recorded health care data, collected without knowledge of our research aim, reduced the risk of information bias.

Our study was limited by lack of information on the indication for collecting the blood or LRT specimen. The no infiltrate and no bacteraemia patient group may thus have included people with chronic LRTI or other pulmonary diseases who had pneumococci cultured *e.g.* as part of a diagnostic process in the absence of any acute infection, resulting in an overestimation of the relative risk of adverse outcomes in the other groups. However, all patients in our no infiltrate and no bacteraemia group had to have at least one new or increased pulmonary symptom together with CRP >50mg/L and/or leukocytes >8.8*10^9^/L, and our sensitivity analysis using even stricter CRP and leukocyte cut points showed robust results.

The validity of our estimates depends on *S. pneumoniae* being the causative agent, as well as on accurate division into manifestation groups based of chest X-ray findings and blood cultures. For the purpose of this study we included patients with chest X-rays describing a “likely” or “probable” infiltrate in the infiltrate without bacteraemia or bacteraemia groups. Assuming that patients with a likely infiltrate are less ill than patients with a definite infiltrate, any misclassification is unlikely to create a false difference between the infiltrate and no infiltrate patient groups, and would not change our conclusions. Results of sputum culture may be falsely positive due to upper airway colonization with pneumococci [36-38]. Nevertheless, colonization is more common in young children and patients with chronic pulmonary disease, and excluding the latter group did not alter our risk estimates notably.

Because the study was done in a routine clinical setting, microbiology tests and chest X-rays were only obtained at the managing physician’s discretion. Thus, not all patients had all tests done. Some patients in the two non-bacteraemic groups might have had bacteraemia, and some patients in the no-infiltrate group might have had an infiltrate without our knowledge. However, this would likely lead to bias towards the null, and our sub-analysis excluding patients with no X-ray or blood culture taken did not change our findings markedly.

## Conclusions

With the above limitations in mind our study demonstrates that hospitalization with pneumococcal LRTI is a severe disease even without presence of pulmonary infiltrate or bacteraemia.

Still, there is a substantial and clinically important outcome gradient from LRTI patients with pneumococcal isolation only to those with concomitant pneumonia and bacteraemia, which is at least in part mediated by higher acute disease severity and inflammation. Though pneumococcal culture results are not usually available at time of admission and thus not suitable for initial clinical decision-making, the results of the present study may foster our understanding of disease mechanisms and the clinical course of pneumococcal disease. Positive chest X-ray findings add important prognostic information, and a positive blood culture predicts substantially increased mortality, higher risk of ICU admissions and pulmonary complications, and longer length of stay.

## Abbreviations

BMI: Body Mass Index; CI: Confidence Interval; CRP: C-Reactive Protein; CT: Computed Tomography; DNRP: Danish National Registry of Patients; ICD-10: International Classification of Diseases, 10^th^ revision; ICU: Intensive Care Unit; IQR: Interquartile Range; LOS: Length Of Stay; LRT: Lower Respiratory Tract; LRTI: Lower Respiratory Tract Infection; MAP: Mean Arterial Pressure; MRR: Mortality Rate Ratio; SAT: Peripheral arterial oxygen saturation.

## Competing interests

H. C. Schønheyder is co-inventor of a patent for an adjuvant of conjugated pneumococcal vaccine. The remaining authors declare that they have no competing interests.

## Authors' contributions

MS participated in the design of the study, collected and analyzed data and drafted the manuscript. HCS participated in the design of the study, organized the gathering of pneumococcal samples at Aalborg Department of Clinical Microbiology, and helped to draft the manuscript. JDK and CØ organized the gathering of pneumococcal samples at Hvidovre Department of Clinical Microbiology. JB organized the gathering of pneumococcal samples at Herlev Department of Clinical Microbiology. RBN helped with the statistical analysis. TB participated in the design of the study and coordinated clinical data collection. H-CS, LL and HBK managed the pneumococcal database and organized the serotyping. RWT participated in the design of the study, and helped to draft the manuscript. All authors were involved in interpreting data and critically revised the manuscript, and all authors read and approved the final manuscript.

## Pre-publication history

The pre-publication history for this paper can be accessed here:

http://www.biomedcentral.com/1471-2334/13/197/prepub

## Supplementary Material

Additional file 1 **Flowchart.** A detailed chart review was done for 1,169 episodes of pneumococcal isolation from the blood and/or lower respiratory tract in patients 15 years or older. Patients were excluded if they did not comply with our predefined study criteria. Thus the final study cohort consisted of 705 hospitalized patients with pneumococcal LRTI. LRT, lower respiratory tract; LRTI, lower respiratory tract infection; CRP, C-reactive protein. Click here for file

Additional file 2 Method of LRT-sampling according to LRTI manifestation group. Click here for file

Additional file 3 ICD-10 discharge diagnoses according to LRTI manifestation group. Click here for file

Additional file 4 CRP and leukocyte counts according to LRTI manifestation group, stratified by age groups. Click here for file

Additional file 5 Outcomes according to LRTI manifestation group overall and for the four supplementary analyses. Click here for file
