# Supplementary material for: Impact of positive chest X-ray findings and blood cultures on adverse outcomes following hospitalized pneumococcal lower respiratory tract infection: a population-based cohort study
Source: BMC Infect Dis. 2013 May 2;13:197. doi: 10.1186/1471-2334-13-197 (PMC3655859; doi:10.1186/1471-2334-13-197)
Supplement: Additional file 1 — Flowchart. A detailed chart review was done for 1,169 episodes of pneumococcal isolation from the blood and/or lower respiratory tract in patients 15 years or older. Patients were excluded if they did not comply with our predefined study criteria. Thus the final study cohort consisted of 705 hospitalized patients with pneumococcal LRTI. LRT, lower respiratory tract; LRTI, lower respiratory tract infection; CRP, C-reactive protein. [file 1471-2334-13-197-S1.pdf]

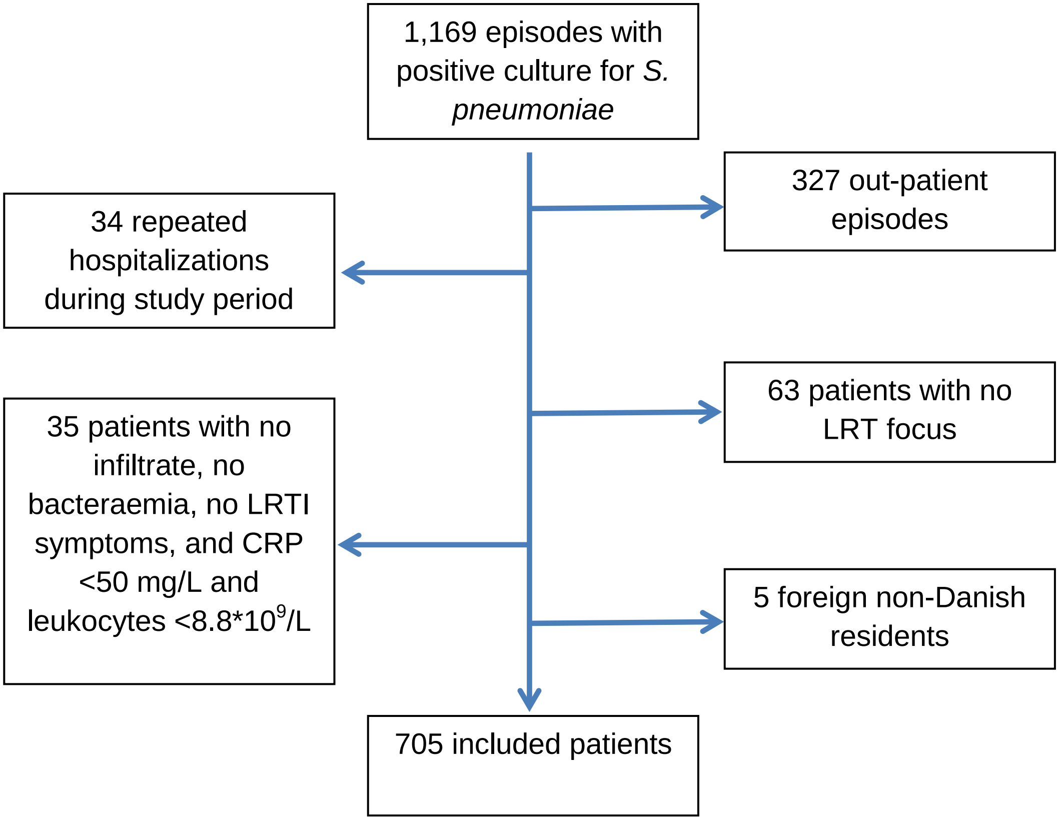

1,169 episodes with positive culture for *S. pneumoniae*
327 out-patient episodes
34 repeated hospitalizations during study period
63 patients with no LRT focus
35 patients with no infiltrate, no bacteraemia, no LRTI symptoms, and CRP <50 mg/L and leukocytes <8.8*10^9/L
5 foreign non-Danish residents
705 included patients
