# Supplementary material for: Impact of positive chest X-ray findings and blood cultures on adverse outcomes following hospitalized pneumococcal lower respiratory tract infection: a population-based cohort study
Source: BMC Infect Dis. 2013 May 2;13:197. doi: 10.1186/1471-2334-13-197 (PMC3655859; doi:10.1186/1471-2334-13-197)
Supplement: Additional file 2 — Method of LRT-sampling according to LRTI manifestation group. [file 1471-2334-13-197-S2.pdf]

## Additional file 2 – Method of LRT-sampling according to LRTI manifestation group

| | No infiltrate and no bacteraemia patients | Infiltrate without bacteraemia patients | Bacteraemia patients | Total | Total of those with a LRT sample obtained |
|---|---|---|---|---|---|
| | n (%) | n (%) | n (%) | n (%) | n (%) |
| Method not specified | 0 (0) | 0 (0) | 1 (0) | 1 (0) | 1 (0) |
| Sputum | 177 (92) | 204 (82) | 67 (26) | 448 (64) | 448 (80) |
| Blind endotracheal suction | 10 (5) | 43 (17) | 32 (12) | 85 (12) | 85 (15) |
| Bronco alveolar lavage | 0 (0) | 1 (0) | 13 (5) | 14 (2) | 14 (3) |
| Pleural fluid | 6 (3) | 2 (1) | 1 (0) | 9 (1) | 9 (2) |
| LRT sample not obtained | 0 (0) | 0 (0) | 148 (56) | 148 (21) | - |
| Total | 193 (100) | 250 (100) | 262 (100) | 705 (100) | 705-148 = 557 (100) |

LRTI, lower respiratory tract infection; LRT, lower respiratory tract
