# Supplementary material for: Impact of positive chest X-ray findings and blood cultures on adverse outcomes following hospitalized pneumococcal lower respiratory tract infection: a population-based cohort study
Source: BMC Infect Dis. 2013 May 2;13:197. doi: 10.1186/1471-2334-13-197 (PMC3655859; doi:10.1186/1471-2334-13-197)
Supplement: Additional file 3 — ICD-10 discharge diagnoses according to LRTI manifestation group. [file 1471-2334-13-197-S3.pdf]

Additional file 3 – ICD-10 discharge diagnoses according to LRTI manifestation group

	No infiltrate and no bacteraemia patients	Infiltrate without bacteraemia patients	Bacteraemia patients	Total
Discharge diagnosis	n (%) – nCPD [a]	n (%) – nCPD [a]	n (%) – nCPD [a]	n (%)
**Discharge diagnosis of acute LRTI**	107 (55) - 69	181 (72) - 88	198 (76) - 51	486 (69)
DA403 (Pneumococcal bacteraemia) [b]	1 (1) - 0	2 (1) - 1	58 (22) - 12	61 (9)
DJ13 (Pneumococcal pneumonia)	9 (5) - 4	39 (16) - 17	43 (16) - 6	91 (13)
DJ180 (Bronchopneumonia)	1 (1) - 1	1 (0) - 1	0 (0) - 0	2 (0)
DJ181 (Lobar pneumonia)	0 (0) - 0	1 (0) - 1	2 (1) - 0	3 (0)
DJ188 (Other pneumonia unspecified)	0 (0) - 0	1 (0) - 1	0 (0) - 0	1 (0)
DJ189 (Pneumonia unspecified)	38 (20) - 18	101 (40) - 38	82 (31) - 25	221 (31)
DJ20 (Acute bronchitis)	2 (1) -1	0 (0) - 0	1 (0) - 0	3 (0)
DJ22 (Acute LRTI, unspecified)	1 (1) - 1	5 (2) - 2	3 (1) - 0	9 (1)
DJ440 (COPD with acute LRTI)	18 (9) - 14	8 (3) - 8	1 (0) - 1	27 (4)
DJ441 (COPD with acute exacerbation)	37 (19) - 30	23 (9) - 19	8 (3) - 7	68 (10)
**No acute LRTI discharge diagnosis**	86 (45) - 43	69 (28) - 26	64 (24) - 7	219 (31)
Total	193 (100) - 112	250 (100) - 114	262 (100) - 58	705 (100)

LRTI, lower respiratory tract infection; CPD, Chronic Pulmonary Disease as defined in the Charlson Comorbidity Index (ICD-8: 490-493, 515-518. ICD-10: DJ40-DJ47, DJ60-DJ67, DJ68.4, DJ70.1, DJ70.3, DJ84.1, DJ92.0, DJ96.1, DJ98.2, DJ98.3).

[a] Data are n (% of all in the manifestation group) – number (n) of patients with CPD in the manifestation group with the respective ICD-10 diagnosis.

[b] Only patients with concomitant LRTI symptoms and/or pulmonary infiltrate were considered in this group.

For all patients we reviewed their ICD-10 discharge diagnoses to see if they had one of the above diagnoses as their primary diagnosis. For those who did not have a primary acute LRTI diagnosis, we looked if they had an acute LRTI diagnosis as their secondary diagnosis. If they had neither, they we placed in the category “no acute LRTI discharge diagnosis”.
