# Supplementary material for: Impact of positive chest X-ray findings and blood cultures on adverse outcomes following hospitalized pneumococcal lower respiratory tract infection: a population-based cohort study
Source: BMC Infect Dis. 2013 May 2;13:197. doi: 10.1186/1471-2334-13-197 (PMC3655859; doi:10.1186/1471-2334-13-197)
Supplement: Additional file 4 — CRP and leukocyte counts according to LRTI manifestation group, stratified by age groups. [file 1471-2334-13-197-S4.pdf]

Additional file 4 - CRP and leukocyte counts according to LRTI manifestation group, stratified by age groups

		No infiltrate and no bacteraemia patients [a]	Infiltrate without bacteraemia patients [a]	Bacteraemia patients [a]
CRP (mg/L)				
	**All patients**	**82 (36-140)**	**163 (70-246)**	**316 (219-404)**
	15-49 years	110 (50-157)	112 (62-223)	314 (247-434)
	50-74 years	71 (32-148)	162 (78-252)	315 (216-414)
	≥75 years	96 (51-131)	190 (80-249)	317 (192-388)
Leukocyte count (10^9/L)				
	**All patients**	**14 (11-17)**	**14 (11-19)**	**17 (12-23)**
	15-49 years	14 (10-20)	14 (12-16)	17 (12-23)
	50-74 years	14 (11-18)	15 (11-20)	16 (11-22)
	≥75 years	15 (11-16)	14 (11-19)	18 (13-23)

LRTI, lower respiratory tract infection; CRP, C-reactive protein
[a] Data are median (interquartile range)
Results were available for >87% of the patients.
