# Supplementary material for: Impact of positive chest X-ray findings and blood cultures on adverse outcomes following hospitalized pneumococcal lower respiratory tract infection: a population-based cohort study
Source: BMC Infect Dis. 2013 May 2;13:197. doi: 10.1186/1471-2334-13-197 (PMC3655859; doi:10.1186/1471-2334-13-197)
Supplement: Additional file 5 — Outcomes according to LRTI manifestation group overall and for the four supplementary analyses. [file 1471-2334-13-197-S5.pdf]

Additional file 5 – Outcomes according to LRTI manifestation group overall and for the four supplementary analyses

	No infiltrate and no bacteraemia patients			Infiltrate without bacteraemia patients			Bacteraemia patients		
	n (%) [a]	Crude RR [b]	Adjusted RR [c]	n (%) [a]	Crude RR [b]	Adjusted RR [c]	n (%) [a]	Crude RR [b]	Adjusted RR [c]
30-day-mortality									
**All patients**	**9 (5)**	**1.0 (ref.)**	**1.0 (ref.)**	**27 (11)**	**2.4 (1.1-5.0)**	**1.9 (0.9-4.1)**	**54 (21)**	**4.8 (2.4-9.6)**	**4.1 (2.0-8.5)**
Sub analysis 1 [d]	9 (5)	1.0 (ref.)	1.0 (ref.)	16 (8)	1.4 (0.6-3.2)	1.3 (0.5-3.0)	45 (20)	4.1 (2.0-8.4)	3.5 (1.6-7.3)
Sub analysis 2 [e]	3 (4)	1.0 (ref.)	1.0 (ref.)	15 (11)	3.1 (0.9-10.5)	2.6 (0.7-9.4)	40 (20)	5.7 (1.8-18.6)	4.5 (1.4-14.9)
Sub analysis 3 [f]	2 (3)	1.0 (ref.)	1.0 (ref.)	23 (12)	5.0 (1.2-21.3)	4.3 (1.0-18.8)	46 (20)	8.3 (2.0-34.1)	7.4 (1.8-31.2)
Sub analysis 4 [g]	3 (3)	1.0 (ref.)	1.0 (ref.)	15 (8)	3.0 (0.9-10.0)	2.4 (0.7-8.7)	31 (16)	5.9 (1.8-19.8)	6.2 (1.9-20.8)
LOS (days)									
**All patients**	**5 (2-9)**	**1.0 (ref.)**	**1.0 (ref.)**	**6 (4-16)**	**1.6 (1.3-2.0)**	**1.5 (1.2-1.9)**	**8 (4-19)**	**2.0 (1.6-2.4)**	**2.1 (1.7-2.6)**
Sub analysis 1 [d]	5 (2-9)	1.0 (ref.)	1.0 (ref.)	6 (4-15)	1.6 (1.3-2.0)	1.5 (1.2-1.9)	8 (5-19)	2.0 (1.6-2.4)	2.0 (1.6-2.6)
Sub analysis 2 [e]	5 (2-16)	1.0 (ref.)	1.0 (ref.)	8 (3-21)	1.8 (1.3-2.6)	1.8 (1.2-2.5)	8 (5-20)	1.9 (1.4-2.6)	2.0 (1.5-2.8)
Sub analysis 3 [f]	5 (3-8)	1.0 (ref.)	1.0 (ref.)	6 (4-17)	1.7 (1.2-2.2)	1.6 (1.1-2.1)	9 (5-20)	2.1 (1.6-2.8)	2.3 (1.8-3.1)
Sub analysis 4 [g]	4 (2-8)	1.0 (ref.)	1.0 (ref.)	6 (4-12)	1.6 (1.3-2.1)	1.5 (1.2-1.9)	8 (5-18)	2.2 (1.8-2.8)	2.4 (1.9-3.0)
ICU admission									
**All patients**	**14 (7)**	**1.0 (ref.)**	**1.0 (ref.)**	**51 (20)**	**2.8 (1.6-5.0)**	**2.4 (1.4-4.1)**	**60 (23)**	**3.2 (1.8-5.5)**	**3.1 (1.8-5.5)**
Sub analysis 1 [d]	13 (8)	1.0 (ref.)	1.0 (ref.)	37 (17)	2.3 (1.3-4.2)	2.0 (1.1-3.6)	48 (22)	2.8 (1.6-5.1)	2.7 (1.5-5.0)
Sub analysis 2 [e]	7 (9)	1.0 (ref.)	1.0 (ref.)	38 (28)	3.2 (1.5-6.9)	2.7 (1.3-5.6)	47 (23)	2.7 (1.3-5.7)	2.7 (1.3-5.6)
Sub analysis 3 [f]	6 (8)	1.0 (ref.)	1.0 (ref.)	42 (23)	2.9 (1.3-6.6)	2.3 (1.0-5.2)	58 (25)	3.2 (1.4-7.2)	3.2 (1.4-7.1)
Sub analysis 4 [g]	5 (5)	1.0 (ref.)	1.0 (ref.)	30 (17)	3.5 (1.4-8.9)	2.5 (1.0-6.3)	44 (22)	4.8 (1.9-11.7)	5.0 (2.1-12.1)
Pulmonary complications [h]									
**All patients**	**2 (1)**	**1.0 (ref.)**	**1.0 (ref.)**	**12 (5)**	**4.6 (1.0-20.5)**	**4.6 (1.0-21.4)**	**36 (14)**	**13.3 (3.2-54.5)**	**12.6 (2.9-53.8)**
Sub analysis 1 [d]	1 (1)	1.0 (ref.)	1.0 (ref.)	8 (4)	6.5 (0.8-51.5)	6.6 (0.8-57.9)	31 (14)	23.9 (3.3-173.8)	22.0 (2.9-167)
Sub analysis 2 [e]	2 (2)	1.0 (ref.)	1.0 (ref.)	10 (7)	3.0 (0.7-13.3)	3.2 (0.7-14.7)	29 (14)	5.8 (1.4-23.6)	5.7 (1.4-24.0)
Sub analysis 3 [f]	1 (1)	1.0 (ref.)	1.0 (ref.)	9 (5)	3.8 (0.5-29.4)	3.7 (0.4-30.9)	34 (14)	11.3 (1.6-81.3)	10.6 (1.4-77.8)
Sub analysis 4 [g]	0 (0)	1.0 (ref.)	1.0 (ref.)	4 (2)	-	-	31 (16)	-	-

LRTI, lower respiratory tract infection; RR, relative risk; LOS, length of stay; ICU, intensive care unit; ref, reference; CI, confidence interval.
[a] Data are median (interquartile range) or n (% of all in the manifestation group).
[b] The crude measures are median ratios (95% CI), prevalence proportion ratios (95% CI) or MRR (95% CI).
[c] The multiple adjusted relative risks are computed using cox-regression for the 30-day-MRR, linear regression for LOS, and Poisson regression for ICU admission and pulmonary complications. The estimates are adjusted for age, sex, smoking, alcohol, and comorbidity.
[d] Restriction to patients who all had CRP >100mg/L or leukocytes >10*109/L and at least one new or increased pulmonary symptom.
[e] Restriction to patients without chronic pulmonary disease.
[f] Restriction to patients who have all had chest X-rays and blood cultures taken.
[g] Restriction to patients with an ICD-10 discharge diagnosis of acute LRTI (ICD10 codes: J13, J18.0, J18.1, J18.2, J18.8, J18.9, J20, J20.2, J20.9, J22, J44.0, J44.1).
[h] Pulmonary complications include pleural effusion, pleural drainage, empyema, and lung abscess.
Data available for over 99%.
